# Functional outcome and Health-related Quality of life two years after lobectomy and sublobectomy for early stage non-small cell lung cancer: A New Center Experience

**DOI:** 10.1186/1749-8090-10-S1-A29

**Published:** 2015-12-16

**Authors:** Ashraf Ali Mohammed Ali Elshorbagy, Yasser Shaban Mubarak, YasserAli Kamal

**Affiliations:** 1 Department of Cardiothoracic Surgery, El-Minia University Hospital, Al-Minia Governorate, Al-Minia City 61519, Egypt

## Background/Introduction

Choice of sublobectomy remains controversial in patients with early non-small cell lung cancer (NSCLC).

## Aims/Objectives

To evaluate the impact of lobectomy and sublobectomy on health-related quality of life (HRQoL), and pulmonary function tests (PFTs) after 2 years of follow-up in patients with stage I NSCLC.

## Method

The study included 82 patients underwent lobectomy or sublobectomy for stage I non-small cell lung cancer, all of them completed two years follow-up and respond to a short form 36 (SF-36) questionnaire at out-patients clinic of a new unit of cardiothoracic surgery, Al-Minia University Hospital, Egypt, between January 2010 and December 2014. Changes in forced expiratory volume in one second (FEV1) and forced vital capacity (FVC) were analyzed

## Results

Among the studied patients, 56 underwent lobectomy and 26 underwent sublobectomy. Patients in both surgical methods were similar regarding age, gender, height, presence of COPD and preoperative pulmonary function tests (PFTs). When compared to sublobectomy at the end of follow-up, patients with lobectomy had significant decline in median of FVC (observed: 1.9 L vs. 2.65 L, p < 0.001; and % predicted: 53.8% vs. 78.64%, p < 0.001) and FEV1 (observed: 1.78L vs. 2.33 L, p < 0.001; and % predicted: 68.5% vs. 83.79%, p < 0.001). There was insignificant difference in overall score and in isolated 8 domains of SF36 (physical functioning, physical role, bodily pain, general health, vitality, social functioning, emotional role and mental health) between both groups. Preoperative FEV1 and FVC were significantly correlated with overall HRQoL.

## Discussion/Conclusion

Sublobectomy results in better preservation of pulmonary functions than lobectomy for management of stage I NSCLC, with no significant difference in HRQoL two years after resection. Sublobectomy should be considered for patients with preoperative poor PFTs.

